# New Home, New You: A retrospective mixed‐methods evaluation of a health‐related behavioural intervention programme supporting social housing tenants

**DOI:** 10.1111/hex.13700

**Published:** 2023-01-11

**Authors:** Martha Paisi, Zoe Allen, Jill Shawe

**Affiliations:** ^1^ School of Nursing and Midwifery University of Plymouth Plymouth Devon UK; ^2^ Peninsula Dental School University of Plymouth Plymouth Devon UK

**Keywords:** adult, health, housing, mental health, social housing

## Abstract

**Background:**

Social housing tenants are at greater risk of engaging in unhealthy behaviours than the general population. Housing association employees are in an ideal position to contribute positively to their tenants' health. ‘New Home, New You’ (NHNY) is a joint venture between a social housing association, a city council and a community healthcare provider in the South West of England. It was designed with the aim of improving the health and well‐being of social housing tenants.

**Objectives:**

The aim of this retrospective evaluation was to establish whether social housing tenants were benefiting from this health‐related behavioural intervention in terms of their mental well‐being and health‐related behaviours.

**Methods:**

This was a mixed‐methods evaluation. The outcome evaluation was a secondary analysis of quantitative data collected during the NHNY project. The process of delivering and receiving the intervention was evaluated using qualitative, semi‐structured interviews with housing officers and tenants who had participated in the programme. The development of the intervention was evaluated through a focus group and additional semistructured interviews with key stakeholders. Quantitative data were analysed using the Statistical Package for the Social Sciences. Qualitative interviews were analysed using thematic analysis.

**Results:**

Six key stakeholders and a total of seven housing officers from several teams and seven tenants were interviewed. Of the 1016 tenants who were invited to participate in NHNY, 226 enroled in the programme. For participating tenants, the scope for health‐related behaviour change was greatest in relation to eating and smoking. Small positive statistically significant changes in mental health were found between the 6‐ and 12‐month mean score and between the baseline and the 12‐month score.

**Conclusions:**

The findings indicate that NHNY may have been beneficial for some participating tenants. Housing officers can have a significant role in promoting health messages and embedding behaviour change among their tenants. Although the programme was implemented as a service improvement rather than a controlled trial and focused on a particular intervention and geographical area, other housing associations may find this evaluation useful for considering whether to adopt some of the principles applied in NHNY in other settings.

**Patient or Public Contribution:**

A social housing tenant representative provided input on the methodology and methods used to evaluate NHNY, as well as the information sheet.

## 1 INTRODUCTION

Health outcomes are strongly related to the conditions in which people are born, grow, live, work and age.^1^ Evidence shows that people from low‐income groups and those residing in deprived areas are more likely to experience poor health outcomes and have a lower life expectancy.^2^, ^3^ For example, in a large US study which analysed data on income and mortality from 1999 through 2014, the gap in life expectancy between the richest 1% and poorest 1% of individuals was 14.6 years for males and 10.1 years for females.^4^ A social gradient in health can also be seen for health‐related behaviours, with people of lower socioeconomic status (SES) being more likely to eat unhealthily, be sedentary and smoke, compared with those of higher SES.^2^ Furthermore, although there has been a general increase in the prevalence of poor mental health, those of lower SES are disproportionately affected.^5^

Social housing tenants are more likely to have lower SES and to report financial hardship, therefore being made more likely due to the social determinants of health to engage in unhealthy behaviours than the general population.^6^ It has been demonstrated that they have higher levels of chronic conditions and health risk factors, including smoking and sedentary lifestyles, compared with those living in other forms of housing.^7^ In addition, they are 1.5 times more likely to experience poor mental health.^8^ Both physical and mental health are important to overall well‐being, while the effect of well‐being on health is substantial and comparable to other risk factors such as an unhealthy diet.^9^

Given the potential benefits that health‐related behaviour changes can bring to individual health and public health, it is no surprise that health research devotes considerable time to identifying effective behavioural interventions.^2^ Considering the morbidity and mortality associated with chronic health conditions, the importance of addressing modifiable health‐related behaviours in high‐risk populations has been highlighted.^6^ Promoting behaviour change among people at the lower spectrum of income is considered a major means of achieving a reduction in health inequalities.^2^

Although limited by its observational nature, previous research has identified an association between housing, the built environment and aspects of mental health and well‐being. Housing associations can contribute substantially to the health of their tenants.^10^ There are various ways in which housing can impact well‐being, for example, through providing a property in good material condition, or through proving a trusted, central organization, a tenant can approach if they need help, advice or support. Assured tenancy with a social landlord can also give people new hope and the opportunity to focus on improving their health by changing their health‐related behaviours. People in the housing provider workforce, especially those supporting people living in social housing, have opportunities to speak with new and current tenants about their health and well‐being and support them to make behavioural changes.^11^ Although, there is some evidence to suggest that gaining a tenancy in affordable and appropriate accommodation has a positive impact on health and well‐being, this association is complex.^12^ This may reflect both the limitations of current evidence and the complexity of the relationships between housing and well‐being for vulnerable people with complex needs.^12^ To date, there is little academic literature to evidence the impact of public health intervention in social housing settings^12^ and a paucity of research specific to residents of social housing.^6^ An evidence review on housing associations and housing interventions^10^ concluded that there is a need for ‘evidence of the health and wellbeing impacts of housing associations' community‐centred work to be produced and published’ to develop the evidence base in this field.

While there are many health‐related behaviours that influence a person's health and well‐being, including sleep and social contact, the ‘One You’ campaign^13^ focuses on the four behaviours of exercise, diet, drinking (alcohol) and smoking, for which there is robust evidence that they influence health outcomes and people's well‐being. Evidence demonstrates that people who are involved in decisions about their health are more satisfied with the services they receive and feel that the decisions made were the most appropriate for their circumstances.^14^ Thus, involving individuals in decisions about their health can ensure that people make informed decisions about their behaviours.

Plymouth Community Homes (PCH) is the largest social housing landlord in Plymouth city and provide homes to over 35,000 people in the area. ‘New Home, New You’ (NHNY) is a joint venture between PCH, Plymouth City Council and Livewell Southwest. The programme has been designed with the aim of improving the health and well‐being of PCH customers. Applicants for PCH properties will have been waiting for a suitable home for any number of years or months. Often those waiting will have been living in properties that are unsuitable for them, such as being overcrowded, not adapted to their disabilities or even with no home at all. Being given a home for life that meets people's needs is a good opportunity for them to decide to make other positive changes to their lives. Recognizing that housing association employees have a unique role in engaging with people at this moment in their lives,^11^ there was an opportunity to assist with the city‐wide health and well‐being agenda by ‘Making Every Contact Count (MECC)’^15^, ^16^ and training PCH staff to provide them with the tools to help people make those changes. Thus, the NHNY project was established. This approach aligns with the Ottawa Charter's principles of Health Promotion, namely strengthening community actions and developing personal skills.^17^ The project supports new tenants and transferring tenants to consider and achieve self‐set health and well‐being goals, through interaction with suitably trained housing officers.

### 1.1 Aim

The aim of this evaluation was to establish whether social housing tenants were benefiting from a health‐related behavioural intervention in terms of their mental well‐being and health‐related behaviours (moving, smoking, alcohol consumption and eating) and whether the intervention was delivered and received as intended. As this was a pilot intervention, the evaluation also investigated the wider strategic learning about developing the intervention.

### 1.2 Staff training

All housing officers and some managerial staff at PCH received training as part of the NHNY project. A 3‐h training session was developed and delivered by Livewell Southwest, 6 months before the launch of the NHNY project. The content was specific to Plymouth city and the NHNY project and aimed to develop skills in motivational interviewing, health considerations and signposting to suitable support services and opportunities in the community and to national resources. All housing officers (including any new staff) received a refresher training session from Livewell Southwest, 18 months after the first training (12 months after the launch of NHNY). Internal staff training about the process and monitoring of NHNY was provided to all staff before the start of the NHNY project, and ongoing support with the administration was given to staff throughout.

### 1.3 Programme delivery and monitoring

Participation in the programme was entirely voluntary and offered at the time of sign‐up to a standard tenancy (as distinct from housing with support, provided for older people with additional needs). If tenants agreed to take part in the NHNY project, they were offered a one‐to‐one conversation with their housing officer, who had received the health and well‐being‐related training.

Before this meeting, their current health and well‐being were assessed using two questionnaires. The two data collection tools used were the Short Warwick‐Edinburgh Mental Wellbeing Scale (SWEMWBS)^18^, ^19^, ^20^, ^21^ and the ‘How Are You’ (HAY) quiz.^22^ The housing officer then explored with the new resident whether there was anything they would like to improve in relation to smoking, diet, physical activity, alcohol consumption and/or mental well‐being and if so, provided the tenant with support to achieve it using goal setting and motivational interviewing. Thereafter, the housing officer suggested ways to improve health and well‐being. They also signposted people to appropriate organizations for help and advice relating to their goals.

Tenants also received a welcome pack upon moving into their new homes. In addition to the useful household items included in PCH's usual welcome pack (pack of tea, coffee, tea cloth and other items), NHNY participants also received well‐being‐related items such as toothbrushes and toothpaste, vouchers for replacing smoking with e‐cigarettes (where relevant), along with information about health and well‐being services. In addition to this, participating tenants received a fortnightly, free, home‐delivered bag of fresh vegetables for 3 months. PCH also provided free cookery sessions for those who wish to learn how to cook healthy meals using the vegetables provided as part of the project. The housing officer then met with the tenant at 1 month and after 6 months and then visited or telephoned them at 12 months for a catch‐up and to complete the two questionnaires again.

## 2 METHODOLOGY

### 2.1 Theoretical framework

The programme theory for NHNY draws upon the ‘behaviour system’ of ‘capability, opportunity and motivation’^23^ and involves:

(1) Education (provision of information to improve capability and motivation)
(2) Persuasion (motivational interviewing to increase motivation)
(3) Incentivisation (enhanced ‘Welcome Pack’ on moving in and fortnightly vegetable bag delivery to improve motivation)
(4) Training (cooking lessons to improve capability)
(5) Enablement (access to resources to improve capability, motivation and opportunity).

### 2.2 Design

The University of Plymouth was asked to evaluate the impact of the intervention retrospectively. This was a mixed methods study design, which included a quantitative and qualitative methodology.

The three aspects of the evaluation were:

#### 2.2.1 Outcome evaluation

This was a retrospective evaluation, which analysed data collected during the NHNY project. All analysed data had already been collected and were provided by PCH, following the signing of an agreement policy between the University of Plymouth and PCH. The University was not involved in the choice of the NHNY data collection tools. Consent to participate and share data anonymously in any written report was gathered before the time of data collection by the housing officer. It was agreed that all data would be held securely and kept in line with the PCH data storage and protection policies and that they would be used to examine the impact of the project.

Participants in this data set signed up to NHNY between the start of the programme (23rd October 2017) and 31st December 2018. The sample consists of all NHNY participants who agreed to share their data.

The survey outcomes for health‐related behaviours were gained through the completion of the HAY quiz.^22^ The HAY quiz was chosen by NHNY stakeholders because it functions as an interactive tool to promote improvement in health‐related behaviours for those completing it, as results are accompanied by specific advice. It was also publicly available and easily accessible. The HAY quiz enables the assessment of an individual's health‐related behaviours with regard to eating, alcohol, smoking and exercising.

The SWEMWBS was chosen to assess the mental well‐being of participants throughout the programme, and it is a validated tool for measuring the change in intervention studies.^18^, ^19^, ^20^, ^21^ The short version includes seven more rigorously tested statements each of which describes a positive state of well‐being and is rated by participants on a 5‐point Likert scale.

#### 2.2.2 Process evaluation

The process of delivering and receiving the intervention was evaluated using qualitative, semi‐structured interviews with housing officers and tenants who had participated in the programme. Interviews also touched upon reported behaviour changes, as part of housing officers' and tenants' experiences of the programme.

Housing officers were invited after a presentation by the evaluation team on a staff away day. Tenants were invited by their housing officer after completion of the programme, as the evaluation was conducted retrospectively and researchers did not have direct access to tenants' contact details. This was followed up by a telephone conversation with the interviewer (Z.A.) to answer tenants' questions and arrange the interview. Participants provided their informed consent in writing before participating.

The semistructured interviews were conducted face‐to‐face (Z.A.). Housing officers were interviewed in private meeting rooms on PCH premises during their working days. Tenants were interviewed in their own homes or at a suitable location on university premises, at their preference. Tenants were provided with a £30 voucher in recognition of their time spent participating in the evaluation. Topic guides were developed to guide the interviews with tenants and housing officers. All interviews were digitally recorded and transcribed by a staff member at PCH. Interviews took place between October 2019 and February 2020, immediately pre‐COVID‐19 pandemic.

#### 2.2.3 Formative evaluation

The development and implementation of the intervention were evaluated through a focus group and additional semi‐structured interviews with key stakeholders who had been involved from the outset. Potential participants were invited by email and were provided with a participant information sheet. Participants provided their informed consent in writing before participating. The focus group (Z.A. and M.P.) and telephone interviews (Z.A.) followed a qualitative approach, allowing participants to respond to general questions, based on a topic guide, in their own words. The focus group and interviews were digitally recorded using a digital audio recorder and transcribed by a staff member at PCH following the signature of a confidentiality agreement.

### 2.3 Data analysis

The Statistical Package for the Social Sciences (SPSS, version 24) was used for the analyses of quantitative data (M.P.). Continuous and categorical variables are presented as means (standard deviation [SD]) and frequencies (%), respectively. Listwise deletion was applied for missing information. The normality of data was tested before statistical tests were conducted. A *p*‐value of less than .05 was considered to suggest statistical significance.

Interview transcripts were uploaded onto NVivo 12 software. Data were analysed using thematic analysis as described by Braun and Clarke.^24^ Verbatim transcripts were coded line‐by‐line and key themes and subthemes were developed (Z.A.). Coding decisions and themes were interrogated (Z.A., M.P., J.S.) and adjusted part way through and at the end of the analysis, thus ensuring rigour in analysis. All transcripts were checked for accuracy against the original recordings and corrected where necessary by Z.A.

## 3 FINDINGS

**Figure 1:**
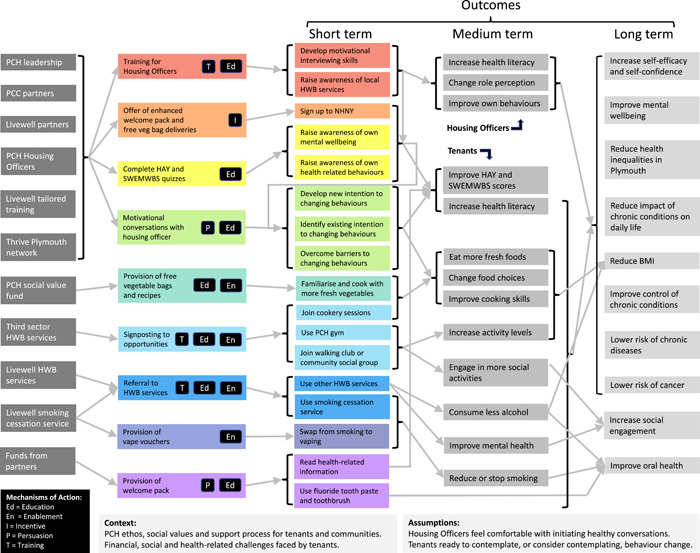
New Home New You: A logic model

A logic model showing all intended inputs, outputs and outcomes is shown below (Figure 1). This was initially developed during the evaluation planning process and was updated after taking into account information gathered from people who participated in the evaluation.

### 3.1 Outcome evaluation

**Figure 2:**
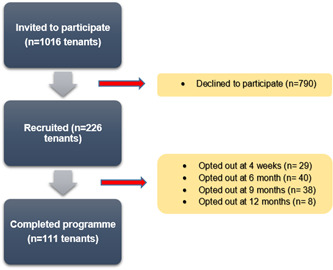
Participant flow diagram

Figure 2 below presents the flow of participants during NHNY.

Of the 1016 individuals who were invited to participate in NHNY (these would have been named as main tenants), 226 signed up for the intervention, yielding a response rate of 22.2%. Of these, 111 completed the intervention. Therefore, the programme was limited in its reach, and the retention of participants was low.

#### 3.1.1 Demographic characteristics

The baseline results below (Table 1) and those at Months 6 and 12 refer to the 111 participants who completed the NHNY programme. The column in grey shows the characteristics of those who dropped out (*n* = 115).

**Table 1:** Baseline characteristics of NHNY participants

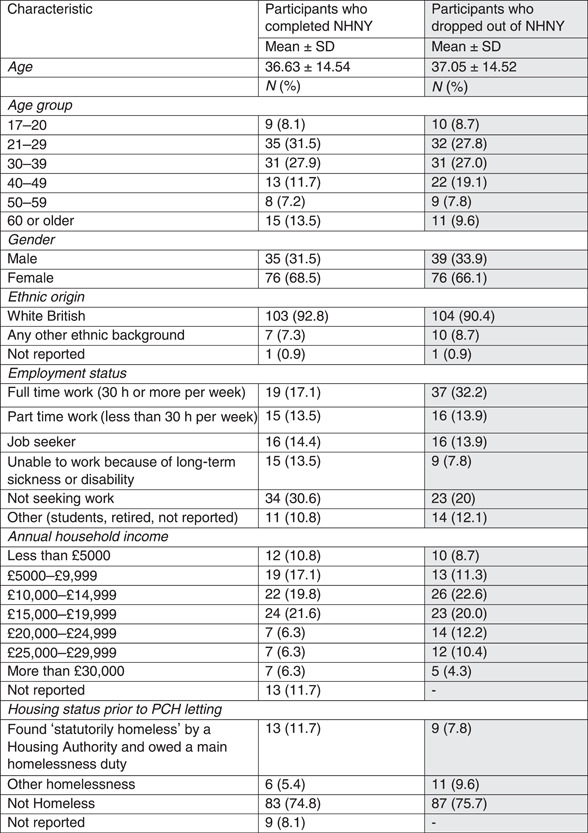

Abbreviations: NHNY, New Home, New You; PCH, Plymouth Community Homes; SD, standard deviation.

The average age of the participants who completed the NHNY programme was 36.6 years, which was similar to the mean age for all eligible tenants (38.9 years) and younger than the overall PCH tenant population (52.1 years). There were 35 (31.5%) males and 76 females (68.5%), which is similar to the split of gender among those who were invited (35.7% male: 63.2% female) and the overall PCH tenant population (39.3% male; 60.5% female and 0.2% unknown). The majority of the participants completing NHNY identified as White British (*n* = 103, 92.8%), reflecting the low ethnic diversity in the city (93%).^25^ The percentage of White British among the overall PCH tenant population was 78.4%. The age and ethnicity of those recruited to the intervention may differ from the overall tenant population due to the inclusion of tenants of housing with support within the overall figures.

Comparing people who completed the programme with those who dropped out, there were no significant differences in proportions in terms of gender (*χ* ^2^ (1) = 0.145; *p* = .703), age (*χ* ^2^ (5) = 3.106; *p* = .684), ethnic group (*p* = .609—*exact test*) or annual household income (*χ* ^2^ (7) = 5.615; *p* = .585).

#### 3.1.2 HAY quiz

Most frequently, participants who completed the programme scored category 2 at baseline, which is considered ‘low’. A Wilcoxon, two‐tailed test comparing the baseline median score to the 6‐month median score showed that there was a positive change which was statistically significant (*Z* = −2.915, *p* = .004). The same test indicated significantly statistically significant positive changes between the 6‐month median score to the 12‐month one (*Z* = −3.663; *p* < .001) and between the baseline and the 12‐month score (*Z* = −5.563; *p* < .001).

#### 3.1.3 SWEMWBS

Baseline SWEMWBS results were recorded for 103 of the NHNY participants who completed the programme, with an average (mean) transformed score of 23.28 (SD 4.23), which was not significantly different from the England average (mean 23.5; SD 3.90).^18^

A paired *t*‐test (two‐tailed) showed that there was an increase of .001 in the score at 6 months compared to the baseline, which was not statistically significant (*t*_95_ = 0.002; *p* = .999). The same test indicated statistically significant positive changes between the 6‐month mean score (23.56) and the 12‐month score (24.74) (mean difference: −1.18; *t*_98_ = −2.451; *p* = .016) and between the baseline (23.29) and the 12‐month score (24.51) (mean difference: −1.22; *t*_99_ = −3.425; *p* = .001).

### 3.2 Process evaluation

A total of seven housing officers from several teams were interviewed. Seven tenants also participated in interviews, including four women and three men. Their ages ranged from early 30s to mid‐60s and all identified as being of White British ethnicity. Only one tenant interviewed was raising a young child.

The following key themes were identified in the participants' interviews:

(1) Challenges faced by tenants
(2) Tenants' perceptions of the value of their tenancy
(3) Housing officers' perceptions of their roles
(4) Staff training
(5) Invitation to participate
(6) Implementation
(7) Effects on tenants
(8) Staff perceptions of programme delivery

This section summarizes information about these themes and includes selected quotes from a range of housing officers and tenants who were interviewed, for illustration.

#### 3.2.1 Challenges faced by tenants

Housing officers and tenants reported that PCH tenants may be experiencing financial constraints, health issues, vulnerability or deprivation when they begin a tenancy. Housing officers mentioned financial constraints, health inequality, vulnerability and deprivation. Tenants often reported health issues.

We were pleased we had got in. We thought, we have got a roof over our heads, we are alright, we can manage. (Tenant)

I think the biggest impact has been Universal Credit really … people can get themselves into a lot of trouble with the rent really quickly, due to that. (Housing Officer)

#### 3.2.2 Tenants' perceptions of the value of their tenancy when facing challenges

Tenants often described how beneficial their new PCH tenancy was for their physical and mental health and social circumstances, independent of their participation in NHNY.

…I just thought when I moved in here it would be like moving into anywhere else I moved into … but it's not like that … they look after you. It makes you feel safe…. (Tenant)

Tenants linked these positive impacts to the way they were treated as tenants, the assurance of a long‐term tenancy and the suitability of their PCH home to their needs. PCH tenancies had released tenants from difficult circumstances in their previous accommodation, such as overcrowding or being confined to unsuitable accommodation due to impairments.

Older tenants, with experience renting privately, appreciated the security of feeling settled in a ‘forever home’ (Tenant).

…in private [rented accommodation] … you can't make it your own home. Where we can make this our own home. (Tenant)

#### 3.2.3 Housing officers' perceptions of their roles

Recognizing the challenges and inequalities faced by their tenants, most housing officers described having a role to play in helping tenants to manage or overcome these issues, which were frequently linked to suitability for a tenancy or maintaining the tenancy. The social work of a housing officer had actively drawn several people to take up the role and was perceived to be valued by PCH. Some also talked about the communication skills they used to tackle sensitive issues with tenants in the course of their work.

…I work with social services, the schools, the police, we do a lot of joint working … There is a lot of social work, I will find, within the [housing officer] role…. (Housing Officer)

Some took a holistic approach to support tenants, whilst recognizing there were limits to the time that could be committed and that some tenants will not engage. Others felt that health and well‐being was a different type of work, which they were not qualified to do and which would be intrusive upon all tenants.

…there's always been about level of thinking about someone's health, but this has moved beyond just that, this has moved to like, what can we do to really try and improve someone's general wellness and how they get on with their lives…. (Housing Officer)

#### 3.2.4 Staff training

Some housing officers felt their colleagues talked more about mental health and about wanting to change their health‐related behaviours since receiving NHNY training and some reported making changes. Others were already aware of their less healthy behaviours but had not been motivated by the training to make changes.

…it is an eye opener, to be perfectly honest it is, but have I actually done anything about it? … maybe, maybe somewhere in the back of my mind it's stuck with me. (Housing Officer)

Some housing officers had become aware of local health and well‐being services to which they could signpost tenants, because of the training. Some felt the focus on motivational interviewing skills would help colleagues who were uncomfortable broaching personal issues with tenants; though some of the housing officers interviewed felt they personally had this ability already.

#### 3.2.5 Invitation to participate

Housing officers reported that the sign‐up process had changed because it was time‐consuming and housing officers were uncomfortable with asking people questions about health‐related behaviours. The process moved online, with tenants completing the health‐related behaviour questions after the first meeting.

…it's morphed so that we send out this email asking people if they are interested and that has the links on it. (Housing Officer)

Housing officers suggested tenants may decline because they are not interested, or they have a lot to do linked to moving home. Housing officers indicated that completing online quizzes may be a barrier affecting capability, especially for older tenants and people who do not have digital devices.

Some housing officers felt there were no trends, demographically, in who joined the programme. One housing officer suggested that tenants' intentions to change their health‐related behaviours may affect their motivation to participate.

It depends on someone's time of life as well, doesn't it, whether they are wanting to make a change or not, you know. (Housing Officer)

Tenants whose lives were being impacted by chronic health conditions described the programme as an opportunity to obtain support to improve their current health situation.

Housing officers reported that, for some tenants, access to vegetable bags helped to mitigate food poverty. Most tenants interviewed signed up for the free vegetable bag deliveries, with some signing up for this reason alone.

They just said you get a free pack of veg every couple of weeks, so I thought well, why not? Don't look a gift horse in the mouth. (Tenant)

#### 3.2.6 Implementation

Some tenants and housing officers viewed the programme as providing people with support to make changes, without receiving criticism. Housing officers found that over several visits they could build up trust and create opportunities to go into more depth with health‐related conversations.

However, housing officers reported that completing the quizzes and conversations was time‐consuming alongside their usual procedures.

…when we do a pre‐tenancy assessment it takes an hour at least, just to go through that, and then to add the New Home New You onto that as well, it was quite a lot…. (Housing Officer).

Staff were aware that the programme tailed off and tenants lost interest when the free vegetable bag deliveries stopped. Visits were sometimes replaced by phone calls, and it could be difficult to contact people for follow‐up.

They also felt that worry and poor mental health were barriers to participation.

…there are people that are very low mood and how you can support them is another thing again really. (Housing Officer)

Staff suggestions included offering the programme to other tenant groups, increasing staff interaction and tangible support and improving digital access. Tenants who completed the programme were generally happy with it, suggesting only that the vegetable bags could include more variety and extend for a longer time.

#### 3.2.7 Effects on tenants

Most tenants reported greater awareness of the health impacts of their behaviours. Some had changed their perceptions regarding their health and their capability to influence it. Several reported that they had lost weight and some had noticed existing health conditions and pain had improved. Some felt their relatives had also benefited.

…because I'm cooking healthier, he's eating healthier and he's quite happy, he's lost a bit of weight as well and he's quite pleased about it…. (Tenant)

All tenants interviewed appreciated the vegetable bags, which had led to sustained healthier food purchases and meal preparation for those who felt their diet could be improved. A few participants felt the programme had no impact other than being a source of free vegetables.

Few tenants actively discussed mental health, though several described feeling more settled since moving home. Tenants reported they had engaged in walking, exercise classes or using a gym, which they had maintained afterwards. Some reported a social or mental health benefit to getting outdoors however some were constrained by physical health or finances.

Housing officers reported that participants who smoked were often interested in stopping and they felt well prepared to assist with vouchers or referrals to smoking cessation services. Tenants who were interviewed and who smoked did not use these services. Tenants and housing officers reported that drinking a lot of alcohol was rare.

#### 3.2.8 Staff perceptions of programme delivery

The housing officers who were most enthusiastic about the programme saw it as an opportunity to establish rapport with tenants, to understand their world and preempt problems. However, some housing officers felt hypocritical about promoting health‐related behaviours that they did not follow themselves.

Housing officers often reported that there was a fine balance between offering to support tenants and intruding upon their personal lives.

…it also felt a little bit intrusive, because some of the questions are really personal, particularly the mental health sort of side of things… (Housing Officer)

Housing officers frequently reported feeling uncomfortable about the language used in the HAY quiz, to the extent that quizzes had become an online task. In addition, they found that response options did not always reflect tenants' behaviour changes.

Most housing officers valued feedback on the programme's impact on tenants, as this made them feel their efforts to engage tenants were worthwhile.

### 3.3 Formative evaluation

Six key stakeholders participated in describing the prerequisites which enabled them to develop and implement the NHNY programme and the challenges encountered in the early stages.

#### 3.3.1 Connections, leadership and social values

The leaders described having a vision for improving health and reducing inequalities to increase people's life chances. They had the power and influence to initiate interventions, which enabled leaders to act upon organizational values of being ‘more than just bricks and mortar’ (Stakeholder). This was seen as pivotal to developing and investing in the programme.

…it was always felt that when someone moves into a new home … it was the perfect opportunity for them to make a change… (Stakeholder)

#### 3.3.2 Challenges of defining and resourcing the programme

It took time to agree on the scope of the programme and the roles of different organizations, due to differing priorities around the programme's purpose, emphasis and development process. This frustrated some stakeholders.

…there was a process at the start where we had lots of meetings … about what we were going to do, was it ethical, would it do any harm and that seemed for us to drag on quite some months… (Stakeholder)

The cost of vegetable bag deliveries, welcome packs, housing officer's time and the time spent devising and delivering training, were absorbed by stakeholder organizations.

#### 3.3.3 The success of utilizing the moment of change

NHNY was considered to enable staff to support tenants at a key moment of change, making the best use of existing staff contacts with new tenants. It was felt to have the potential to increase housing officers' capability and motivation to review their own health‐related behaviours.

#### 3.3.4 Staff perceptions and motivation

Initially, stakeholders found that staff perceptions of their role and motivation to deliver NHNY varied considerably. By seeking staff feedback and acting upon suggestions, stakeholders adapted NHNY to make it more acceptable and feasible for housing officers to deliver the programme.

#### 3.3.5 Dedicating staff time

Allowing time for staff training, engaging tenants and delivering NHNY appeared to be critical to programme delivery but also at a premium, particularly for initial staff training.

…a lot of praise has to go to the [external training] team … who did put together a bespoke training programme for the housing officers. (Stakeholder)

#### 3.3.6 Monitoring and recording participant data

Stakeholders had worked through numerous challenges around what to measure, how to record data and managing the administrative burden of the programme. Stakeholders decided to provide incentives (free vegetable bags) to overcome possible barriers to participation linked to the formal consent process.

## 4 DISCUSSION

Our findings indicate that although limited in its reach, NHNY may have been beneficial for some participating tenants, possibly creating an opportunity and enhancing capability for health‐related behaviour change, particularly for participants who were already internally motivated to change. The current evaluation has identified a number of factors influencing participant engagement and acceptability as well as housing officer engagement. Housing associations may adopt some of the principles applied in NHNY in other settings to promote their tenants' well‐being.

Our evaluation has shown statistically significant improvements in some health outcomes of some participating tenants. However, only 22.2% of tenants who were invited took the opportunity to participate in the NHNY programme. Whilst staff did not report any clear trends in their interview responses, some of the more engaged tenants who were interviewed reported that significant pre‐existing physical health issues had contributed to their interest in participating in the programme. Of those who accepted the invitation, approximately 49% completed the programme at 12 months. This is in line with other research which demonstrated low retention rates with vulnerable adults.^26^ The timing and complexity of the NHNY sign‐up process appeared to be a barrier to participation for tenants. Proactive methods to contact participants (e.g., text messaging) could help improve follow‐up rates.^26^ More broadly, ‘a continuously dynamic process of monitoring intervention progress and tailoring strategies to particular circumstances’, has been recommended for maximizing retention.^26^, ^27^

The mental well‐being scores for NHNY participants compare well with the England average,^18^ despite the recent adversity experienced by some tenants reported by tenants and housing officers. Overall, there appears to be potential to improve equity of access to support with mental health and health‐related behaviour change by adapting the programme design to enable people who are experiencing poorer mental health or who are not ready to contemplate behaviour change to engage with support from housing officers. It, therefore, appears prudent as per the principles of MECC,^15^, ^16^ to embed conversations about health in general housing officer activity so that tenants have opportunities to engage without having to formally contemplate and consent. Given the complex interplay of factors affecting mental well‐being, consideration should be given to the ways in which housing associations can connect with external agencies such as mental health support, the police and social services to provide such support to their tenants.^5^

Given that participants frequently commented on the value they placed on the vegetable bags, this was clearly a significant component of the NHNY intervention as a whole. Interview data suggested this may have been beneficial to participants on several levels, leading to sustained healthier food purchases and meal preparation for those who felt their diets could be improved. Considering that lack of access to affordable and nutritious foods are common barriers to healthy eating among tenants in public housing,^28^, ^29^ social housing providers should consider whether free vegetable deliveries could form part of an organization's existing support process for new tenants experiencing food poverty, without obligation to sign up to a programme.

The evaluation showed that there was a statistically significant improvement in the overall HAY quiz score over the course of the NHNY programme, for those who completed the programme. When viewing individual changes in HAY scores, an overall improvement in individual participants' scores was seen in 43.9% of participants. The finding that effects were not uniform across the participants is consistent with existing literature. The scope for health‐related behaviour change was greater in relation to eating and smoking. Similarly, in a mixed‐methods study involving public housing residents, smoking among residents was found to be extremely high, while only 22% and 29% of 88 participants reported consuming more than one serving of fruit or one serving of vegetables, respectively, per day.^6^ Considering that human self‐regulation draws on limited resources, interventions may be more effective when they focus on one behaviour change at a time,^2^ ideally the one chosen by the tenants themselves.

There was also a statistically significant improvement in reported mental well‐being among participants by the end of the NHNY intervention. The Warwick Medical School guidelines indicate that this change of 1.22 points is borderline clinically meaningful. Although this positive small change could well be attributed to NHNY, we cannot exclude the possibility that other factors contributed to this. Housing may facilitate positive changes in the mental health of permanent supported housing tenants.^30^ However, in many cases, there is limited, or lack of evidence of the effect of housing interventions on well‐being and effects are not necessarily uniform across groups of vulnerable people.^12^ Some of the participants interviewed after the NHNY programme described these positive feelings in relation to the sense of security and suitability that their overall PCH tenancy provided. Owing to the design of the intervention, it is not possible to ascertain the extent to which the NHNY programme contributed to the positive outcome changes, independent/in addition to this sense of security. A project adopting a randomized controlled design would provide the strongest evidence that improvement in outcomes was attributed to the intervention, although it is recognized this design is difficult to achieve for community‐based health promotion interventions.

Owing to being landlords, housing associations could reach people that public health interventions may not otherwise reach.^10^ As was the case with NHNY, housing officers can make important contributions to the well‐being of their tenants and may be involved in the delivery of services.^10^ PCH staff varied in their willingness to engage in conversations about health with tenants. This appeared to relate to their varying perceptions about the boundaries of the housing officer role and feeling a sense of intrusion if asking about tenants' behaviours before establishing rapport and trust, which is in line with perceived challenges among other housing associations' staff.^11^ A cultural shift, whereby staff understand the benefits of such programmes, is therefore particularly important in similar contexts.^11^ For housing officers to reach their full ‘public health potential’, effective training (e.g., understanding of public health, and behaviour change techniques) must be provided.^11^

The administration of the programme, which some housing officers perceived to be onerous, and the language used in the HAY quiz, which made them feel uncomfortable, appeared to de‐motivate housing officers, even when they were committed to the programme. Housing officers welcomed the simplification of the administrative process and separation of housing officers from the quiz completion process, which may reduce barriers to staff engagement. This highlights the need to involve housing officers in programme development, implementation and monitoring. Such an approach can also enhance a feeling of ownership and also help staff understand a project.^11^ The organizational commitment to NHNY appeared to assist some housing officers to put a greater focus on supporting tenants' and communities' well‐being and opportunities, beyond tenancy maintenance. In fact, organizational readiness is considered an important element for the implementation of MECC,^11^ while strong leadership is imperative in bringing about a cultural change.

### 4.1 Limitations

The generalizability of results to highly urban areas with diverse ethnic groups may be limited. The generalizability of the study is further attenuated by the low response rate and subsequently small sample size. Owing to the design of the study, it is not possible to ascertain through the evaluation the extent to which the NHNY programme contributed to these changes. Although having a control group would have allowed us to control for confounding factors, this was not possible due to the retrospective nature of the evaluation and the design of the intervention. In addition, tenants' responses to the HAY quiz and SWEMWBS, are at risk of bias, as common with other surveys, participants may have been reluctant to be open with housing officers (or with themselves) about their health‐related behaviours.

The HAY quiz is not a validated tool for measuring behaviour change. Therefore, it is difficult to infer what change is clinically meaningful. Furthermore, as also reported by housing officers, the HAY quiz scoring system did not capture all changes that tenants had made to their food consumption or smoking activity, such as switching to vaping.

Lastly, participating tenants were recruited to take part in interviews by their housing officers after completing the programme. It is likely that housing officers would have been more successful in recruiting tenants with whom they had built up a good rapport during the programme. It is also possible that tenants who took part in the interviews were those who were motivated to change their behaviour and may have provided relatively positive feedback. Therefore, the tenants who were interviewed may not be representative of all participating tenants, and the transferability of the findings to those who dropped out or who were not successful in changing their behaviour may be limited. Housing officers who were interviewed may also have had a more positive perception of the programme than those who did not respond to the invitation, and this may have been reflected in their feedback.

## 5 CONCLUSIONS

The findings indicate that NHNY may have been beneficial for some participating tenants. During the pilot phase, many tenants did not take up the opportunity or had limited engagement in the programme. The NHNY pilot has generated key learning points and presents an important opportunity to show how the intervention could be improved. The programme has already been adapted to overcome some barriers to participation and to extend tenants' opportunities to engage in health‐related conversations with housing officers and to access other support services. In particular, to improve uptake, administrative processes have been streamlined. The programme has become more goal‐focused, with tenants defining their chosen goals before receiving free vegetable bags, making it easier for staff to provide appropriate signposting. In addition, measures have been put in place to monitor and evaluate programme outcomes and the effectiveness of the adaptations.

Housing officers can undoubtedly have a significant role in promoting health messages and embedding behaviour changes among their tenants. Although this study focused on a particular intervention and geographical area, other housing associations may find this evaluation useful for considering whether to adopt some of the principles applied in NHNY in other settings. Improved interventions that target health‐related behaviour change among social housing tenants are warranted.

## AUTHOR CONTRIBUTIONS

Martha Paisi, Zoe Allen, and Jill Shawe made substantial contributions to the evaluation conception and design, revised it critically for important intellectual content, gave final approval of the version to be published, and agreed to be accountable for all aspects of the work in ensuring that questions related to the accuracy or integrity of any part of the work are appropriately investigated and resolved. Martha Paisi and Zoe Allen drafted the manuscript and contributed to acquisition and analysis of data.

## CONFLICT OF INTEREST

The authors declare no conflict of interest.

## ETHICS STATEMENT

A confidentiality agreement was put in place between the University of Plymouth and PCH, before data sharing. Both the formative and process evaluation and outcome evaluation were approved by the Faculty of Health and Human Sciences Research Ethics Committee of the University of Plymouth (ref: 18/19‐1147 and ref: 13/14–240, respectively).

## Data Availability

The data that support the findings of this study are available from the corresponding author upon reasonable request.
